# The Supraorbital Keyhole Craniotomy through an Eyebrow Incision: Its Origins and Evolution

**DOI:** 10.1155/2013/296469

**Published:** 2013-07-10

**Authors:** D. Ryan Ormond, Costas G. Hadjipanayis

**Affiliations:** Department of Neurosurgery, Emory University School of Medicine, Atlanta, GA 30322, USA

## Abstract

In the modern era of neurosurgery, the use of the operative microscope, rigid rod-lens endoscope, and neuronavigation has helped to overcome some of the previous limitations of surgery due to poor lighting and anatomic localization available to the surgeon. Over the last thirty years, the supraorbital craniotomy and subfrontal approach through an eyebrow incision have been developed and refined to play a legitimate role in the armamentarium of the modern skull base neurosurgeon. With careful patient selection, the supraorbital “keyhole” approach offers a less invasive but still efficacious approach to a number of lesions along the subfrontal corridor. Well over 1000 cases have been reported in the literature utilizing this approach establishing its safety and efficacy. This paper discusses the nuances of this approach, including the benefits and limitations of its use described through our technique, review of the literature, and case illustration.

## 1. Introduction

Numerous neurosurgical approaches have been developed to operate on lesions of the frontotemporal skull base. These approaches include frontal, bifrontal, frontotemporal, pterional, orbitozygomatic, and other variations [1]. The evolution of these approaches from Dandy's frontotemporal “macrosurgical approach,” to Yasargil's microsurgical pterional approach, and finally to the supraorbital keyhole approach through an eyebrow incision all have served to give the neurosurgeon the exposure they needed to safely address various pathologies [2]. The goal of “keyhole” surgery was not to perform a small incision and craniotomy for the sake of a small opening. The goal of this approach was to permit adequate access to skull base lesions while limiting trauma to surrounding structures such as the skin, bone, dura, and, most importantly, the brain [3–5]. 

The supraorbital craniotomy and subfrontal approach have been used to access a number of pathologies including tumors (meningiomas, craniopharyngiomas, etc.) and vascular abnormalities (e.g., aneurysms, arteriovenous malformations, and cavernous hemangiomas) [1, 2, 5–35]. Surface lesions typically require craniotomies as large as the lesion. Deep-seated lesions, however, can be accessed through a much smaller craniotomy since the intracranial field widens with increasing distance from the skull [2, 3, 5, 36–38]. Utilizing this principle, surgeons can access lesions in the subfrontal, suprasellar, Sylvian fissure, and posterior fossa regions of the brain [2–6, 21]. 

When considering any approach to a pathological entity, it is important to understand the advantages and disadvantages of a given procedure. Surgery through an eyebrow incision may not be appropriate for all lesions of the anterior skull base. There is a narrow viewing angle through this approach that may require frequent adjustment of the operating room table and microscope for adequate visualization of a given lesion. The microscope light is often another problem, as there may be some difficulty getting adequate light through such a small opening onto a deep-seated lesion. Microinstruments require almost coaxial control through such narrow anatomic windows [2, 5]. In the setting of vascular lesions, a smaller opening in a blood-filled field can also make it difficult to obtain adequate vascular control without damage to surrounding structures. 

Use of a rigid rod-lens endoscope in combination with the operative microscope can provide a great benefit with the supraorbital craniotomy and subfrontal approach. The endoscope can provide a much greater light source at the depths of the exposure, with greater focus and better visualization. Ensuring a large enough size to the craniotomy (no smaller than 1.5–2 cm) is important as well to ensure adequate maneuverability of instruments for a bimanual approach to surgery [2, 5]. Through thoughtful consideration of appropriate lesions and adequate experience with this technique, we believe that safe surgery can be performed on numerous pathologies without brain retraction and with a superb cosmetic result.

## 2. Surgical Description

After general anesthesia, endotracheal intubation, and placement of a Foley catheter, the patient is fixed in a Mayfield three-pin head holder with two pins on the ipsilateral posterior cranium and the one pin site on the contralateral frontal bone. The torso is slightly elevated at ten degrees, and the head is positioned in a slightly extended position of around 15–20 degrees to allow gravity retraction of the frontal lobes away from the surgical field. No retractors are used. The head is turned approximately 15–45 degrees contralaterally to the side of surgery to allow appropriate visualization of midline lesions. The bed can be further rotated as necessary for further adjustments during surgery. Midline lesions, such as olfactory groove lesions, require more rotation, whereas laterally placed lesions require less rotation for appropriate visualization and access. The most important information in decision making regarding the side of the approach is the structure of the lesion itself and its relationship to surrounding anatomic structures. Certainly, when either side can adequately access the lesion, we typically choose a nondominant approach in order to reduce the risk of damage to the dominant frontal lobe.

The skin incision is made along the eyebrow without cutting the hair of the eyebrow (Figure 4). Previous studies have shown no increased risk of infection, and leaving the eyebrow intact allows for a better cosmetic result [2, 3, 5, 7, 46]. It is important that the skin incision be placed laterally to the supraorbital notch to avoid forehead numbness from injury to the supraorbital nerve during surgery [2, 5, 21, 22]. The incision is made through the skin and dermis, with dissection continuing superiorly just superficial to the orbicularis oculi, pericranium, and temporalis fascia. Care is taken to ensure that orbicularis oculi fibers are not damaged. This layer is important for closure purposes as well as for an optimal cosmetic result. Dissection continues in this manner approximately 1.5–2 cm superior to the supraorbital ridge. A small retractor can be used to keep the incision open at this point. The pericranium is incised medially beginning lateral to the supraorbital nerve. Pericranial dissection continues in a “C”-shaped fashion extending approximately 1.5–2 cm superior to the supraorbital ridge and laterally to the superior temporal line. This muscle and pericranial flap are reflected inferiorly and retracted out of the way with a suture. 

The craniotomy is made by bluntly dissecting a small portion of temporalis muscle and fascia at the superior temporal line and drilling a 5 mm burr hole on the lateral aspect of the exposure below the temporalis for a better cosmetic result. Care is taken to avoid the use of cautery around the temporalis at this location, as this may cause damage to the frontalis branch of the facial nerve. A craniotome is then used to make two cuts. The first is from the burr hole along the floor of the anterior cranial fossa extending to a position lateral to the supraorbital notch. The second again starts from the lateral burr hole but makes an arch superiorly to then return to meet the medial edge of the first cut. The craniotomy takes the form of a “D,” with the back wall of the “D” along the floor of the anterior cranial fossa. It is important to ensure a craniotomy at least 1.5–2 cm in width, or manipulation of microinstruments is very difficult. It is also important to recognize a breach of the frontal sinus, as this can be a source of CSF leak postprocedure if not adequately addressed. In fact, a very lateral extension of frontal sinus may preclude the use of this approach in a given patient because of the difficulty repairing a large opening in the frontal sinus via this approach. We have used bone wax to seal off any small breach of the frontal sinus and betadine-soaked gel foam to seal off larger defects.

The dura is now dissected off the orbital roof. At this point, the inner table of the inferior edge of the craniotomy is drilled flush with the orbital roof. Any ridges of the orbital roof can also be leveled with the high-speed drill. This not only improves visualization but also allows greater access of instruments during the procedure. A malleable brain retractor may be placed against the dura to protect against unintentional durotomy. The outer table is left intact to maintain cosmesis. Bone dust is washed out with antibiotic irrigation prior to dural opening. The dura is opened in a “C”-shaped fashion and reflected inferiorly with a stitch. The microscope is brought into the field, the frontal lobe is lightly retracted with a cottonoid, and the CSF cisterns are opened to allow CSF egress to facilitate brain relaxation. Following brain relaxation, the primary procedure may be performed safely with no fixed retractors on the brain and with use of the operative microscope, a rigid rod-lens endoscope, or both. 

Wound closure is straightforward. The dural leaflets are reapproximated with a 4-0 Nurolon suture sewn in a running fashion. The craniotomy bone flap is replaced with a titanium burr hole cover and two titanium square plates to improve the cosmetic result by restoring the supraorbital ridge. The pericranium and muscle flap are then closed primarily. Buried, interrupted, and absorbable sutures are used in the dermis, and a 5-0 prolene subcuticular stitch is placed without any knots to ensure removal in the office in 7–10 days. A head wrap can be applied until the first postoperative day to lessen subgaleal edema formation.

## 3. Case Illustrations

A number of case series utilizing this approach have been published in the literature (Table 1). The reported morbidity and mortality in these series are similar to that reported in surgeries on similar pathologies by other approaches. It is important to understand the benefits and shortcomings of this approach so that case selection can be performed appropriately. We have provided a few case examples from our own series to highlight some of the benefits of this approach, as well as ways to make the approach safer and more efficacious using modern techniques, technology, and adaptation.

### 3.1. Case  1

**Figure 1:**
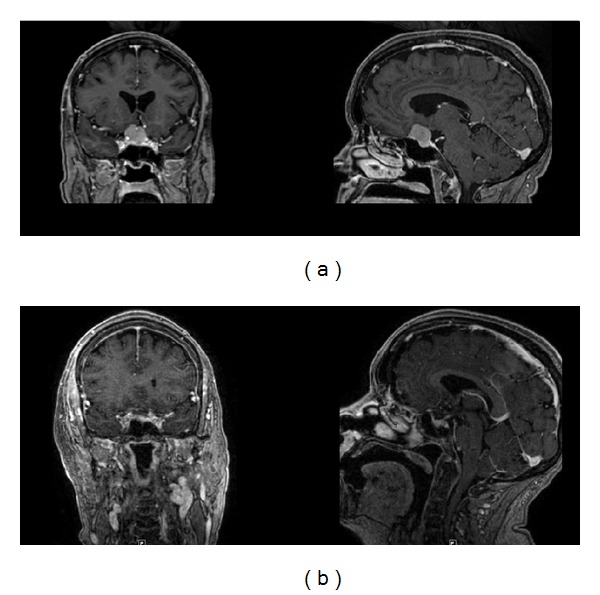
(a) Preoperative and (b) postoperative MR images of a homogeneously enhancing mass involving the tuberculum sellae and planum sphenoidale. Pathology was meningioma. Gross total resection was achieved.

A 71-year-old RH woman presents with a history of progressive headaches who underwent an MRI of the brain with gadolinium contrast administration. The MRI demonstrated a homogeneously enhancing sellar/suprasellar lesion that extended to the planum sphenoidale causing optic chiasmal compression as well as compression of the right optic nerve. The right A2 branch of the anterior cerebral artery coursed through the superior aspect of the tumor. Its imaging characteristics were most consistent with a tuberculum sellae meningioma. This increased in size on subsequent imaging, and the patient underwent elective resection of her tumor by a right supraorbital keyhole craniotomy through the right eyebrow. Preoperative and postoperative imaging are shown (Figure 1). She had a gross total resection of a WHO grade I meningioma and had no visual deficits postoperatively. 

### 3.2. Case  2

**Figure 2:**
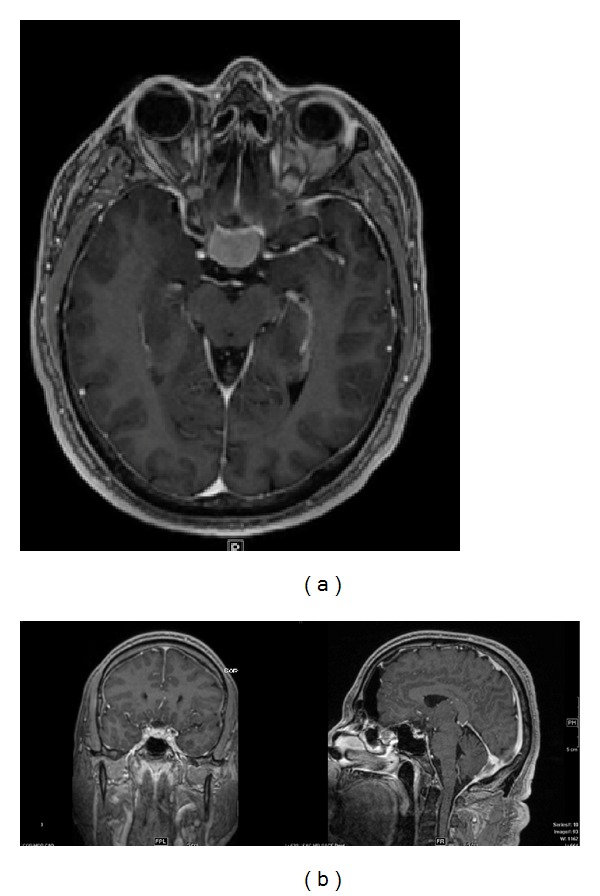
(a) Preoperative and (b) postoperative MR images of a homogenously enhancing mass involving the planum sphenoidale. Pathology was meningioma. Gross total resection was achieved.

A 46-year-old right-handed male presented with visual loss and headache. He could only finger count in the right eye and had an additional bitemporal hemianopsia on visual field testing. Imaging demonstrated a homogeneously enhancing mass involving the tuberculum sellae and planum sphenoidale causing compression of the right optic nerve and chiasm. Imaging characteristics were most consistent with a meningioma. He underwent a right supraorbital craniotomy via an eyebrow incision obtaining a Simpson grade II resection (Figure 2). Postoperatively, his vision improved substantially to where he could read with his right eye and had some improvement in his bitemporal field cut. 

### 3.3. Case  3

**Figure 3:**
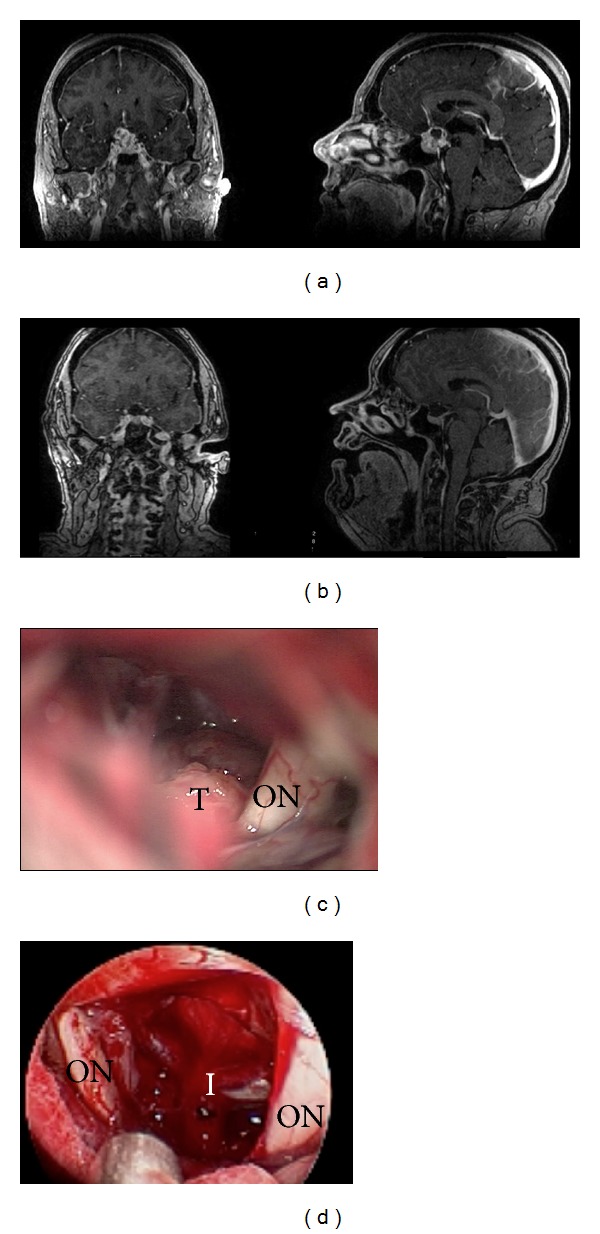
(a) Preoperative and (b) postoperative MR images of a heterogeneously enhancing cystic mass involving the sella and suprasellar region. Pathology was consistent with craniopharyngioma. Near-total resection was achieved. (c) Microscopic images from surgery demonstrate optic nerve (ON) and its relationship to tumor (T). (d) Comparison image from endoscopic view in the same patient now demonstrating both optic nerves (ON) and infundibulum (I) following tumor resection. Note the wider field of view, greater visibility, and contrast at depth. There is also significantly less blur from anatomy obscuring view superficial to focal point as clearly noted in microscopic image (c).

**Figure 4:**
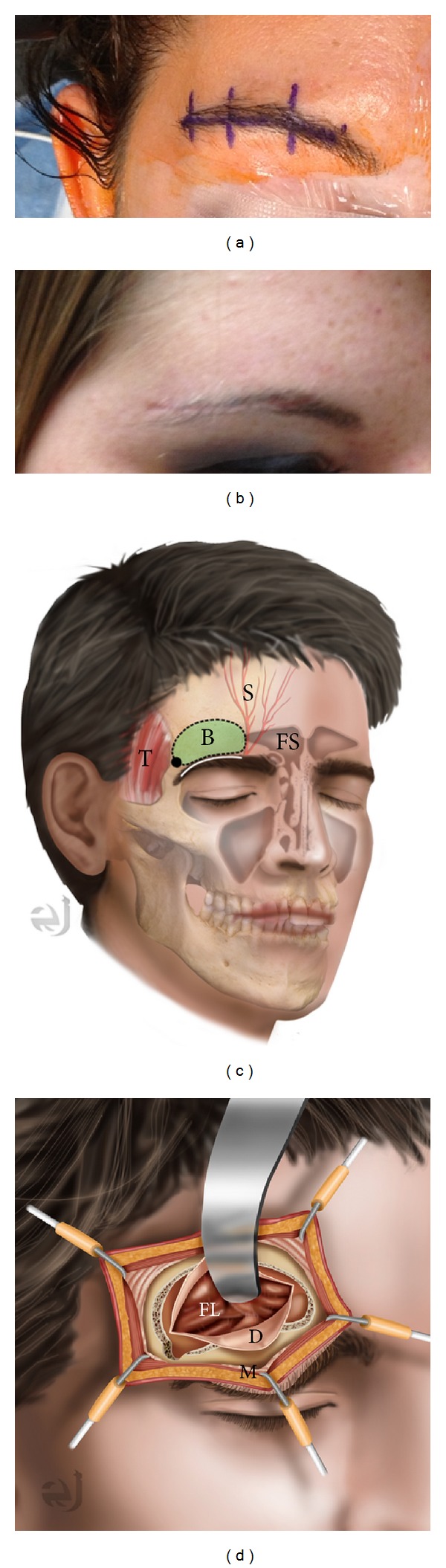
(a) Preoperative image of planned right eyebrow incision and (b) six-week postoperative image in the same patient. (c) Illustration of supraorbital craniotomy through an eyebrow incision. The incision is within the eyebrow (white), lateral to the supraorbital nerve (S) and frontal sinus (FS). The temporalis (T) is separated just posterior to the zygomatic process for the burr hole. Bone flap is approximately 1.5 × 2 cm (B). (d) Illustration after opening demonstrating dural opening (D), retracted frontal lobe (FL). The orbicularis oculi muscle (M) is reflected inferiorly with the pericranium.

A 51-year-old right-handed woman presented with vision loss and headache. Imaging demonstrated a sellar and suprasellar heterogeneously enhancing cystic mass causing optic chiasmal compression (Figure 3). She underwent a right supraorbital craniotomy via an eyebrow incision and had a gross total resection of her craniopharyngioma and preservation of her pituitary stalk (Figure 3). Postoperatively, her vision improved, but she did develop transient diabetes insipidus.

## 4. Discussion

When the supraorbital craniotomy and subfrontal approach through an eyebrow incision were first described, there was significant controversy over the use of this approach in neurosurgery [2, 5]. Many felt that a keyhole approach would limit exposure and not allow adequate visualization to perform safe and successful surgery. Early reports discussed difficulties with cosmesis both from the bony repair and the incision. Postoperative functional loss of the supraorbital nerve or frontalis branch of the facial nerve was common in early case series as well. In the setting of a breach of the frontal sinus, meningitis or CSF leak has also been reported. 

Experience has helped to demonstrate the limitations of the approach, and many of these early limitations have been overcome. A number of case series reported in the literature demonstrate the efficacy of this approach (see Table 1). Gross total resection was achieved in a similar extent as much larger craniotomies, being reported as 89.2% gross total resection of skull base meningiomas in the largest series [2–5]. Morbidity also does not appear to be higher than in other procedures for similar pathologies including a low cerebrospinal fluid (CSF) leak rate (see Table 1). 

### 4.1. Limitations of Supraorbital Craniotomy through the Eyebrow Incision

Entering through the eyebrow historically led to postoperative loss of supraorbital sensation or to a palsy of the frontalis branch of the facial nerve (see Table 1). Placement of the incision lateral to the supraorbital notch is important in preserving function of the supraorbital nerve. Avoiding the use of cautery laterally over the temporalis fascia and muscle can also avoid injury to the frontalis nerve. The use of neuronavigation can help prevent a breach of the frontal sinus during the craniotomy. Avoidance of the frontal sinus will lower the risk of CSF leak or postoperative wound infection. A lateral frontal sinus may even be considered a contraindication for this approach. 

In the setting of vascular pathologies, there may be some difficulty with using two suction tubes in managing prematurely ruptured aneurysms or to obtain proximal control [13, 22, 46, 47]. Some have even recommended against this approach for vascular lesions for this reason [47]. A prominent orbital rim may impede the surgical degree of freedom, and some authors have advocated the addition of an orbital osteotomy to improve surgical freedom and access for vascular pathologies [16, 48]. A similar concept led to similar adaptations to traditional approaches to frontal base and parasellar lesions in the past [46, 49–52]. A number of authors have described different vascular pathologies safely treated through this approach, but we feel it should be limited to those with significant experience with the approach, and it may not be the best approach for some lesions (such as in subarachnoid hemorrhage, giant aneurysms, or vascular lesions in the posterior circulation) in comparison to more traditional approaches (see Table 1) [13, 22, 46, 47]. 

Numerous shortcomings have been overcome since the introduction of this approach in the 1980s. Probably the biggest limitation was the problem of lighting with the operating microscope down such a narrow corridor. Endoscopes have dramatically improved visualization of this region through this approach and allow for safer dissection with better visualization through this smaller incision than can often be achieved with the microscope alone. Endoscopic-assisted surgery is a common adjunct to the modern skull-based surgeon wishing to employ this keyhole approach in his armamentarium, and is discussed in more detail in what follows.

### 4.2. Head Positioning with the Keyhole Supraorbital Craniotomy and Subfrontal Approach

Proper positioning of the head for the keyhole supraorbital craniotomy can play an important role in surgical access of skull base lesions. Extension of the neck permits frontal lobe relaxation in combination with mannitol. Contralateral rotation of the head is also performed [2–5, 9, 13, 48]. The degree of head rotation is related to the anatomic location of the pathology in the subfrontal corridor. One author has recommended 10–15 degrees of rotation for suprasellar lesions and the temporomesial surface, 30 degrees for lesions of the planum sphenoidale, and 45 degrees for lesions involving the cribriform plate [48]. Adjustments in the viewing angle can also be made by rotating the operating room table or by adjusting the microscope or endoscope for appropriate visualization during the procedure. Lumbar drainage is rarely used in any of the case series reported [1, 2, 5–35].

### 4.3. Avoidance of the Supraorbital and Frontalis Nerves

Multiple cadaveric studies have been performed in an attempt to increase the safety of the supraorbital keyhole approach. One study looked at the location and course of the supraorbital nerve and the frontalis branch of facial nerve. This study of ten specimens noted a supraorbital notch in 12/20 sides (right or left) and a supraorbital foramen in the remaining 8 [53]. The lateral branch of the supraorbital nerve has no branches within 10 mm after exiting the supraorbital foramen and notch and courses on the pericranium with an angle with the supraorbital margin of 74 ± 3° (68–80°) [53]. The authors suggest that a more medial craniotomy can be performed without damage to the supraorbital nerve by dissecting below calvarium and elevating pericranium with the supraorbital nerve to expose calvarium for craniotomy without damage to the nerve [53]. Certainly, staying at least 5 mm lateral to the supraorbital notch or foramen with the craniotomy has significantly reduced the risk of supraorbital palsy as well [13, 22, 46]. Incision into the orbicularis oculi should be made along the margin of the muscle superiorly with the muscle dissected with pericranium inferiorly to spare the fibers. The frontalis branch of facial nerve can be injured if the incision extends greater than 13 mm lateral to the zygomatic process of the frontal bone [53]. Therefore, limiting the lateral extension of the incision as well as the use of cautery in the temporalis muscle below the zygomatic process also reduces the risk of frontalis palsy [3, 4, 13, 53]. Finally, another author also recommends sparing the insertion of the temporalis muscle for a better cosmetic result [53]. Using these techniques, among others, has likely played a role in the reduction in supraorbital and frontalis nerve problems in more recent series (Table 1).

### 4.4. Keyhole Approach and Optical Field

An additional cadaveric study sought to quantitatively verify the accuracy of the claims of Perneczky's group that the optical field widened with increasing distance from the keyhole and that contralateral parasellar structures could be visualized well [2–5, 46]. In this study, the supraorbital keyhole approach was compared to the pterional and larger more traditional supraorbital craniotomies. Their findings demonstrate that the difference in area of exposure between approaches was less than 1 cm, and there was no difference in the total or contralateral side area of exposure in the parasellar region between the three approaches [46]. The authors conclude that the limitations in this approach have more to do with “surgical freedom” of microinstruments than in the field of view at depth [46]. Similar results were found in another cadaveric study noting that, for approaching anterior communicating artery aneurysms, the supraorbital keyhole and transorbital keyhole approaches both afforded more area of exposure than the standard pterional approach [54]. 

### 4.5. Supraorbital Keyhole Approach with Endoscopic Assistance

Endoscopes have aided in overcoming one of the main disadvantages to the keyhole approach: illumination. Use of the microscope in keyhole surgery requires frequent changing of the visual angle to allow illumination of the area of interest deep in the surgical field. Endoscopes produce illumination at depth rather than from a distance and therefore can illuminate the area of interest without casting shadows on the field. Endoscopes can be held either by an assistant or with a retractor arm, allowing the surgeon to continue to work bimanually with microinstruments running in a parallel axis with the endoscope [21]. Angled lenses also allow visualization around corners without requiring retraction of important neurovascular structures. This aids in minimizing trauma to the collateral tissue field. A “second look” with the endoscope can also improve the gross total resection of tumors despite the smaller craniotomy with better visualization [21, 22]. The use of angled endoscopes has allowed the supraorbital window to be extended to regions as distant as the interpeduncular cisterns and contralateral cerebellopontine angle by some authors [21]. A secondary advantage to improved illumination with the endoscope is improved ability to achieve hemostasis, which is more difficult through a keyhole approach and listed often as a disadvantage [22]. 

### 4.6. Supraorbital Keyhole Approach for Resection of Tuberculum Sellae Meningiomas in Comparison to Endoscopic Endonasal Extended Approaches

A few case series have been reported regarding both supraorbital keyhole approach or endoscopic endonasal extended approaches for resection of tuberculum sellae meningiomas. One author performed a meta-analysis comparing the endoscopic endonasal extended approach for tuberculum sellae meningioma resection with an open craniotomy approach [55]. In this meta-analysis, abstracts that did not differentiate tumor type and location with outcome were excluded. There were 38 retrospective references, 33 were for open cases and 8 for endoscopic endonasal approaches (3 had both approaches). Results demonstrated a similar rate of gross total resection between approaches (85% versus 84% of open versus endoscopic cases, resp.). However, there was a much higher rate of cerebrospinal fluid (CSF) in the endoscopic cases (26.8% versus 3.5% open cases). This rate differed greatly between endoscopic series, with series utilizing a rigid reconstruction and/or a vascularized nasoseptal flap having a 16% leak rate versus 64% for series with other closure methods. Vision loss was significantly higher for open approaches (9.2% versus 1.3% for open versus endoscopic, resp.), but the open series included much larger tumors, potentially accounting for this difference [55]. Rates of pituitary dysfunction were similarly low across series. Unfortunately, this comparison included multiple types of open approaches and lumped them all together. We were interested in the subset of open series performed through a supraorbital keyhole approach through an eyebrow incision. We performed a MEDLINE search for tuberculum sellae meningiomas similar to Bohman et al. and extracted data on case series that performed surgery through a keyhole approach through an eyebrow incision where outcomes data specific to the location were reported. We found 78 cases reported where this approach was used to resect tuberculum sellae meningiomas (see Table 1) [1, 2, 5–35]. Gross total resections were possible in 67/78 (85.9%) cases. Complications included eight patients with worsening vision, seven with hyposmia/anosmia, one with a corneal abrasion, five with endocrinological problems, and two patients who died (one following ICH from a carotid artery injury, a second from unexplained cardiac arrest 40 days after surgery). There were three CSF leaks and no wound infections. These results are similar to the general open series discussed by Bohman et al., demonstrating no greater risk, with a similar rate of gross total resection, despite the smaller craniotomy [55].

### 4.7. Supraorbital Keyhole Approach for Olfactory Groove Meningiomas

The supraorbital keyhole approach has also been described for resection of olfactory groove meningiomas. In the literature, a MEDLINE search revealed a total of 81 cases reported in the literature where outcomes data were specific to the olfactory groove location of the tumor [1, 2, 5–22, 34]. 74 tumors were resected in a gross total fashion (91.4%). Complications reported included eight CSF leaks and five wound complications. This higher rate of CSF complications may be due to the midline anatomic location of olfactory groove meningiomas. Since the recessed cribriform plate is difficult to visualize with the microscope during a supraorbital keyhole approach, a higher CSF leak rate may occur. Other authors have described an endonasal endoscopic route to these lesions. However, a recent study compared traditional open craniotomy with endoscopic endonasal resection of tumors, concluding that better resections, and lower CSF leak rates, were possible through the open rather than the endoscopic approach [56]. Use of the endoscope for assistance in visualizing the cribriform plate may further permit complete resections of olfactory groove meningiomas while also helping with skull base reconstruction to prevent CSF leakage. 

### 4.8. Supraorbital Keyhole Approach for Resection of Suprasellar Craniopharyngiomas in Comparison to Endoscopic Endonasal Extended Approaches

A case series of 43 patients was recently reported with either craniopharyngiomas or anterior skull base meningiomas resected through either an extended endoscopic endonasal route or through a keyhole supraorbital craniotomy and subfrontal approach [8]. Of the craniopharyngiomas treated, there were 18 treated through an extended endoscopic endonasal approach and 4 treated through a supraorbital route. There was one postoperative CSF leak in the endonasal cohort and none in the supraorbital cohort. There were two gross total resections in the endonasal cohort and none in the supraorbital cohort, although this was often not the goal of surgery. If there were dense adhesions to neurovascular structures, the authors noted they opted for a subtotal resection with planned postoperative radiation [8]. 

The location of the chiasm in relation to the tumor, along with the lateral extension of tumor, may determine whether a supraorbital keyhole or endoscopic endonasal approach is taken. Prechiasmatic craniopharyngiomas may be better accessed through a supraorbital keyhole approach especially if there is lateral or suprachiasmatic extension of tumor. Retrochiasmatic lesions, on the other hand, can pose a greater chance for injury to the visual apparatus through a supraorbital approach and may be better resected through an endoscopic endonasal approach [8]. 

### 4.9. Cosmetic Considerations of the Eyebrow Incision

Cosmesis has prevented many surgeons from attempting this approach or has led to their abandonment of this approach with its introduction early on. A number of modifications have led to what many now consider to be a superb cosmetic result with the supraorbital craniotomy and keyhole approach. A limited skin incision within the eyebrow, minimal temporalis muscle dissection, a small bone flap, and closure with the orbicularis oculi muscle/pericranium layers have contributed to the success of the eyebrow incision. Temporalis muscle atrophy, so common with standard frontotemporal and pterional craniotomies, can be avoided with the eyebrow incision [16]. Of course, orbicularis oculi muscle asymmetry can lead to less ideal cosmetic outcomes through this approach. This can occur through both muscle fiber and nerve injury [24, 25]. This can be avoided by first opening the incision only through the skin and dermis layers, and then opening the muscle more dorsally and cutting along the muscle fibers rather than across them. 

There have been a number of ways to perform the incision including superciliary, transciliary, and even transpalpebral incisions in an attempt to improve cosmesis [6, 9, 24, 26, 29]. Superciliary incisions avoid depilating the hair follicles but leave a visible scar above the eyebrow. Transciliary incisions may lead to hair follicle depilation, but this typically does not occur if one avoids the use of cautery [48]. The transpalpebral approach places the incision through the folds of the eyelid, thus also avoiding depilation of the hair follicles, but typically requires the use of a second specialist with experience performing surgery through the eyelid [26]. All of these incisions can become problematic in the setting of infection, but thankfully infection risk is low with this approach (see Table 1). 

Another important cosmetic consideration is performing the initial incision through the skin and dermis layers only. Cephalad dissection superficial to the orbicularis oculi, pericranium, and temporalis muscle is important for development of a separate tissue flap for covering the keyhole craniotomy during closure [2, 5, 13, 22, 46]. Additional considerations for a good cosmetic result include proper repositioning of the bone flap. Care must be taken to ensure that the outer cortex of the supraorbital ridge remains intact during the approach. Use of a burr hole cover and square titanium plates prevents the appearance or palpation of the gap between the bone flap and intact native bone following bone flap replacement in the patient. Final closure of the skin layer with a running subcuticular stitch (e.g., 5-0 Prolene) without any suture knots brings the edges of the eyebrow together for proper cosmesis as well.

## 5. Conclusions

The supraorbital craniotomy and keyhole approach through the eyebrow permit access to a number of lesions in the subfrontal corridor with minimal brain retraction and a much smaller area of potential injury of superficial structures. All minimally invasive techniques have a learning curve, and smaller, simpler lesions should be performed first through this approach before moving on to larger, more complicated lesions. Our experience is that midline and suprasellar lesions are more easily accessed through this approach than laterally based lesions. Endoscopy can play an important role in improving visualization through the keyhole corridor. Attention to detail can allow this approach to be performed with superb cosmetic results while still achieving surgical efficacy and limiting complications. 

## Figures and Tables

**Table 1:** Case series of keyhole supraorbital subfrontal approaches through an eyebrow incision.

Publication	Year	Patients	Tumors	Aneurysms	Other	Supraorbital hypesthesia	Frontalis palsy	Hyposmia	Wound infection	CSF leak	Hematoma	Perioperative mortality	Diabetes insipidus	Other
Jho [10]	1997	11	11	0	0	11	0	NR	0	0	NR	0	2	Vision worsened (*n* = 2)
Paladino et al. [14]	1998	37	0	40	0	4 (all recovered)	NR	NR	1	NR	NR	0	NR	Aneurysm rupture (*n* = 1)
Van Lindert et al. [21]	1998	139	0	197	0	0	0	0	0	0	0	NR	NR	Aneurysm rupture (*n* = 4)
Sánchez-Vázquez et al. [19]	1999	41	34	6	1	41 (all recovered)	41 (all recovered)	21 (2 permanent)	0	0	0	0	NR	
Czirják and Szeifert [6]	2001	155	52	102	1	2	1	2	2	NR	NR	5	NR	Aneurysm rupture (*n* = 2), PE (*n* = 1)
Dare et al. [7]	2001	10	0	10	0	10 (all recovered)	10 (all recovered)	NR	1	0	0	0	0	Wound edema
Ko et al. [11]	2001	7	3	4	0	4	NR	NR	NR	NR	NR	NR	NR	Periorbital edema (*n* = 7)
Shanno et al. [15]	2001	72	61	0	11	NR	NR	NR	5	5	1	0	NR	Tension pneumocephalus, asp pna, infarct, ICA injury, and corneal abrasion. Lateral rectus palsy (all *n* = 1)
Steiger et al. [16]	2001	33	0	33	0	NR	2	NR	1	NR	NR	0	NR	Diplopia (*n* = 1) resolved
Czirják et al. [29]	2002	36	0	74	0	0	0	0	0	0	0	1	NR	2 intraoperative ruptures
Fernandes et al. [35]	2002	16	10	2	4	NR	NR	1	1	1	0	0	NR	
Ramos-Zúñiga et al. [17]	2002	20	0	20	0	NR	20 (all recovered)	NR	NR	NR	1	0	NR	Blindness (*n* = 1), infarct (*n* = 1)
Wiedemayer et al. [23]	2004	9	7	0	2	1	1	NR	0	0	0	0	NR	
Zhang et al. [39]	2004	54	52	0	2	NR	NR	NR	NR	1	NR	0	NR	
Jallo et al. [9]	2005	28	24	0	4	NR	NR	NR	1	0	0	0	NR	Decreased vision (*n* = 1)
Melamed et al. [12]	2005	25	15	0	10	NR	NR	NR	1	1	0	0	1	Blindness (*n* = 1)
Mitchell et al. [13]	2005	47	0	47	0	1	NR	NR	0	NR	4	1	NR	Aneurysm rupture (*n* = 2), infarct (*n* = 2)
Reisch and Perneczky [2]	2005	450	199	229	22	34	25	27	6	12	4	1	NR	
Lupret et al. [25]	2006	30	0	30	0	10	NR	NR	1	NR	NR	2	NR	Aneurysm rupture (*n* = 4), mucocele (*n* = 2)
Zheng et al. [22]	2007	35	35	0	0	0	0	0	0	NR	0	0	11	
Brydon et al. [28]	2008	50	0	50	0	NR	NR	NR	1	1	1	3	NR	New neurological deficit (*n* = 12)
Fatemi et al. [8]	2009	13	13	0	0	0	2 (both recovered)	NR	0	0	0	0	0	Vision worse (*n* = 1)
Romani et al. [34]	2009	66	66	0	0	0	0	6	4	6	1	0	NR	4 cotton granulomas
Chen and Tzaan [1]	2010	21	5	13	3	NR	NR	NR	NR	NR	NR	2	NR	Hydrocephalus (*n* = 2), infarct (*n* = 1)
Telera et al. [20]	2012	20	20	0	0	NR	NR	6	0	1	1	1	1	4 with worse vision
Abdel Aziz et al. [26]	2011	40	8	31	1	NR	NR	NR	2	1	1	0	NR	Transpalpebral approach, 1 ischemic infarct
Fischer et al. [27]	2011	793	0	989	0	0	0	0	9	9	14	NR independent of other approaches	NR	26 reoperations for inadequate clipping (19 clipping, 7 coiling), 61 intraoperative ruptures
McLaughlin et al. [31]	2011	11	11	0	0	NR	NR	NR	0	1	0	0	0	1 carotid artery injury, 1 bilateral caudate infarcts
Park et al. [40]	2011	13	0	13	0	NR	0	NR	0	0	0	0	NR	All unruptured PCoA aneurysms with CN III palsy, all resolved
Romani, et al. [32]	2011	73	73	0	0	NR	NR	1	1	3	2	3	2	New postop. neurological deficits (*n* = 19)
Chalouhi et al. [41]	2013	47	0	47	0	NR	NR	NR	1	0	1	0	NR	5 intraoperative ruptures, 4 ischemic infarcts
Romani et al. [33]	2012	52	52	0	0	NR	NR	0	0	3	0	0	4	New postop. neurological deficits (*n* = 16)
Ivan and Lawton [42]	2013	2	0	0	2	0	0	0	0	0	0	0	0	Both cavernous malformations
Kang et al. [43]	2013	4	0	4	0	NR	NR	NR	NR	NR	NR	NR	NR	
Ditzel Filho et al. [44]	2013	10	9	0	1	NR	NR	NR	0	0	0	2	NR	Deaths: 1 pulmonary embolus, 1 systemic disease
Park et al. [45]	2013	52	0	52	0	NR	26 (all recovered)	NR	NR	NR	NR	NR	NR	

Totals		2522	760	1993	64	63	57	43	38	45	31	21	21	
Percent of reported (number)		100%	30.5%	79.0%	2.6%	2.8% (53/1881)	3.0% (57/1878)	2.3% (43/1878)	1.6% (38/2364)	2.2% (45/2081)	1.5% (31/2118)	1.4% (21/1527)	8.3% (21/252)	
